# Cavity Swelling of 15-15Ti Steel at High Doses by Ion Irradiation

**DOI:** 10.3390/ma17040925

**Published:** 2024-02-17

**Authors:** Cong Liu, Hailiang Ma, Ping Fan, Ke Li, Qiaoli Zhang, Aibing Du, Wei Feng, Xiping Su, Shengyun Zhu, Daqing Yuan

**Affiliations:** 1 China Institute of Atomic Energy, Beijing 102413, China; congl7271@163.com (C.L.); fanping@ciae.ac.cn (P.F.); zql@ciae.ac.cn (Q.Z.); duaibing401@126.com (A.D.); fengwei1109@sina.com (W.F.); suxiping@cnncmail.cn (X.S.); zhusy@ciae.ac.cn (S.Z.); 2 School of Nuclear Science and Engineering, National Research Tomsk Polytechnic University, 634050 Tomsk, Russia

**Keywords:** 15-15Ti, ion irradiation, irradiation swelling, helium effect, steady-state swelling

## Abstract

The titanium-stabilized austenitic stainless steel Fe-15Cr-15Ni, which shows enhanced resistance to irradiation swelling compared with more traditional 316Ti, has been selected as a core material for fast reactors. Data on the evolution of irradiation swelling in 15-15Ti steels at very high doses, which cannot be easily achieved by neutron irradiation, are still lacking. In this paper, the swelling behavior of the titanium-modified austenitic stainless steel 15-15Ti was investigated by pre-implantation of He at room temperature followed by Ni-ion irradiation at 580 °C to peak doses of 120, 240 and 400 dpa. Relatively small cavities were observed in the zone of helium implantation, while large cavities appeared in the region near the damage peak. A correction formula for the dpa curve was proposed and applied to samples with large swelling. It was found that the steady-state swelling rate of 15-15Ti remains at ~1%/dpa even at high doses. By comparing the swelling data of the helium-implanted and helium-free regions at same doses, 70 dpa and 122 dpa, the suppression of swelling by excessive helium can be deduced at such doses.

## 1. Introduction

Austenitic steels are selected as fast reactor fuel cladding materials because of their excellent high-temperature strength and fine machinability [1,2]. To achieve a high burnup of fuel and boost long-term economic performance for the Sodium-Cooled Fast Reactor (SFR), the dose of fuel assembly could reach up to 200 displacements per atom (dpa) in the future [3]. In the Generation IV SFR program, the integrity of fuel pins is highly dependent on whether the cladding can withstand the high temperature and high burnup irradiation. Swelling remains one of the defining challenges to the structural integrality of claddings in service [4]. Type 316 stainless steel (SS) was chosen as the cladding material for protype fast reactors, but it showed excessive swelling at doses above 50 dpa. It was shown that by adding stabilizing elements, adjusting chemical composition and introducing cold work, swelling can be reduced significantly. The titanium-stabilized austenitic stainless steel 15-15Ti derived from 316 SS exhibits enhanced swelling resistance as a result of its increased nickel content and the formation of intragranular nano-sized TiC precipitates [4,5]. Between 1982 and 1998, 15-15Ti cladding was used in the Phénix reactor, and the maximum damage dose reached 130 dpa. It was reported that the deformation of 15-15Ti is still acceptable. Similar alloys have been selected as core materials for fast reactors, such as ChS-68 (Russia), D9 (USA), D9I (India), Din 1.4907 (Germany) and JPCA (Japan) [6,7].

The swelling of austenitic steels as a function of damage dose is usually characterized by an incubation period in which the swelling is very small or negligible at lower doses, a transient regime and, finally, the steady state of swelling. The nucleation of cavities mainly occurs during the incubation period, resulting in an increase in the number density but no significant change in size [2]. The size of cavities continually increases with accumulating dose, as the generation and absorption rate of vacancies exceeds the emission rate. In the stage of steady-state swelling, the swelling rate is nearly independent of the dose rate, temperature and alloying chemistry for a given material. Theoretical analysis suggested that when dislocations and cavities produced by irradiation were of comparable sink strengths, the separation between interstitials and vacancies was most effective, resulting in steady-state swelling with the maximum swelling rate [8]. The steady-state swelling rate can be used to describe the irradiation resistance of a material [2]. The typical steady-state swelling rates are ~1%/dpa and ~0.2%/dpa for austenitic and ferritic/martensitic steels, respectively [9]. In bcc steels, the lower dislocation bias for preferential absorption of interstitials results in a lower rate of steady-state swelling [10]. 

In-pile irradiation and post-irradiation examination of materials are costly and generally time consuming. The induced radioactivity also makes post-irradiation examinations inconvenient. Heavy-ion irradiation can yield high damage rates without residual radioactivity and at very low cost [11]. Therefore, heavy-ion irradiation is widely used as a surrogate method for neutron irradiation [11,12,13,14,15,16]. Ion irradiations have been critical not only in the research and understanding of radiation effects, but also in the development of innovational materials used in reactors. A great deal of research has been devoted to the understanding of the mechanisms of irradiation-induced swelling using ion irradiation. For example, in Ref. [15], it was demonstrated that the steady-state swelling rate (~1%/dpa) of annealed AISI 304L SS could be reproduced using self-ion irradiation up to ~60 dpa. The swelling rates of two austenitic alloys, cold-worked 316 steel and alloy A709, both reached ~1%/dpa eventually between 200 and 300 dpa using Fe irradiation [16]. However, the evolution of swelling for more irradiation-resistant materials, such as 15-15Ti steel, is limited at very high doses. 

For ion-irradiated materials, extra interstitials introduced by ion implantation will suppress swelling by reducing the cavity nucleation rate when defect recombination is an important process [10]. Such a process would have far-reaching consequences in the more swelling-resistant steels. It was shown in Ref. [17] that far fewer cavities were produced in solution-annealed JPCA irradiated to 50 dpa at 750 and 800 K by heavy ions compared with 316 SS. Cavity-denuded regions can be observed near regions of damage peak [18]. In order to compensate for the swelling suppression of extra interstitials, the implantation of insoluble helium before heavy-ion irradiation was frequently used to enhance cavity nucleation [11,19,20,21]. However, the role of helium, particularly its concentration, is complicated in the evolution of swelling [22,23,24,25,26,27,28,29,30,31,32]. The effect of helium on swelling is believed to depend not only on the dose but also on the helium concentration [27]. It was shown both in cold-worked and solution-annealed austenitic steels that the cavity density tends to increase markedly and that the cavity size decreases gradually with increasing He/dpa ratio [19,27]. The cavity density tends to approach a saturation, while the mean cavity size continues to decrease when the helium concentration exceeds an intermediate He/dpa ratio. In the framework of the dislocation bias model, the swelling is driven by the biased absorption of interstitials and vacancies [25]. The preferential absorption of interstitials by dislocations results in a vacancy supersaturation in the matrix. At the same time, the helium-induced cavity nucleation increases swelling. As the helium concentration continues to increase, the increasing number of cavities, which are the neutral sinks, promotes the recombination of interstitials and vacancies. Therefore, the swelling is hindered. 

Irradiation swelling remains one of the most defining challenges to the use of austenitic steels as high burn-up fuel assembly materials in SFRs. Ion irradiation provides an indispensable tool to study the irradiation effect at high doses which cannot be easily achieved by in-pile irradiation. In this paper, the swelling behavior of 15-15Ti austenitic steels under ion irradiation was investigated at very high doses, aiming to complement the irradiation swelling data and to verify the feasibility of heavy ion irradiation to simulate neutron irradiation in terms of steady-state swelling. Helium implantation prior to ion irradiation was carried out in order to enhance cavity nucleation at low doses and to study the effect of helium on swelling. The irradiation damage region bombarded by heavy-ions can be split into helium-implanted and helium-free regions. The cavity morphology and swelling of both regions were examined by transmission electron microscopy (TEM). Swelling as a function of dpa and the synergistic effect of helium with displacement damage on swelling was discussed. 

## 2. Experimental Procedure

**Figure 1:**
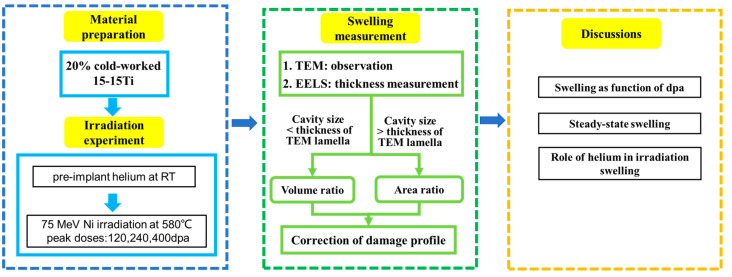
Flow chart of experimental procedure and subjects of discussion. The key steps of this work are highlighted. The blue box shows information about material preparation and irradiation experiments. The green box displays swelling measurements. The yellow box is for discussion.

A flow chart of the experimental procedure and the subjects of discussion is shown in Figure 1. The experimental procedure consisted of material preparation, ion irradiation and swelling measurement. The dpa curves were corrected using the measured swelling data. For details, see the following sections. 

### 2.1. Materials and Irradiation Experiment

The base material used in this paper is a 20% cold-worked 15-15Ti steel. The nominal composition of this steel was given in Ref. [21]. The 15-15Ti steel in the form of a cladding tube was sectioned into Φ15 mm × 1.5 mm disks by electrical discharge machining. The specimens were then mechanically ground and fine-polished to a mirror-like surface prior to irradiation [21].

The irradiation experiments were carried out at the Triple Beam Irradiation Facility in the China Institute of Atomic Energy (CIAE) [33]. The facility consists of an HI-13 tandem accelerator, a 300 kV helium implanter and a 300 kV hydrogen implanter. In the irradiation chamber, the samples were mounted on a copper base and connected to a PBN/PG heater. A sample can be heated from room temperature to 800 °C. A hole was drilled in the middle of the copper base and brazed with a K-type thermocouple to monitor the temperature. A thermostat was connected to the heater and the thermocouple so that the target temperature could be adjusted automatically to a preset value with an accuracy of ±2 °C.

**Figure 2:**
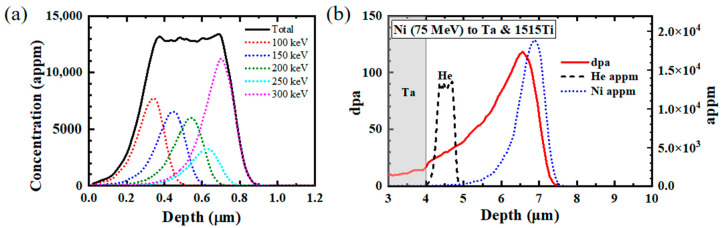
(**a**) Distribution profile of implanted helium. (**b**) Profiles of displacement damage (solid) and implanted Ni (dotted). He implantation profile was drawn with the dashed line.

The specimens were pre-implanted with helium at room temperature before the heavy ion irradiation in order to promote cavity nucleation at low doses. A plateau of helium concentration in the depth zone of 350 to 700 nm under the irradiated surface was created by multiple energy implantation. In a rate theory calculation, the swelling of 15-15Ti will increase with increasing helium concentration and will reach a saturation at a high appm of helium [34]. The maximum helium concentration was set to be 13,000 appm on the plateau. The profile of implanted helium is shown in Figure 2a. Previously, it was observed by TEM that there were no helium bubbles or voids for a similar specimen implanted with helium at room temperature [21]. 

The specimens pre-implanted with helium were irradiated using a 75 MeV defocused Ni beam through a Ta foil of ~4 μm thickness. A defocused beam was preferred to a raster scanning beam, as discussed in the ASTM E521 standard [34,35,36]. The tantalum foil was fixed in front of the sample to reduce the beam energy and to further defocus the beam. 

Swelling only occurs in a certain range of temperatures. If the irradiation temperature is lower, the defects are less mobile and less likely to form larger clusters. At higher temperatures, vacancies can emit from the cavities, which counterbalances the net vacancy flow towards the cavities, limiting growth [25,37]. The maximum swelling occurs at an intermediate temperature, known as the temperature of peak swelling. This temperature is dependent not only on the material but also on the dose rate [25,38,39] and the hydrogen/helium concentrations in multiple-beam irradiations [28]. It will shift to a higher temperature with an increasing dose rate. The temperature of swelling peak in the heavy-ion irradiation of austenitic steels has been measured with various characterization methods, such as positron annihilation techniques [33] and TEM examinations [28]. These studies suggest that this temperature is around 580–590 °C [38] in heavy-ion irradiations generally, with a dose rate in the range of 10^−2^–10^−3^ dpa/s. In this paper, heavy-ion irradiation was performed at 580 °C, the temperature of peak swelling, with peak doses of 120, 240 and 400 dpa. Correspondingly, the doses in the helium implantation zone reached 30, 60 and 122 dpa, respectively. The damage was calculated by the SRIM [40] code using the Kinchin–Pease model, as shown in Figure 2b. The displacement energies of Fe, Cr and Ni were set to 40 eV [41]. 

### 2.2. Swelling Measurement

After the irradiation, TEM lamellas were fabricated by the standard life-out technique using a TESCAN Lyra3 focused ion beam (FIB)(TESCAN, Brno, Czech Republic). TEM observations were carried out by a JEM-2100F microscope (JEOL, Tokyo, Japan). Smaller cavities were observed under the kinematical diffraction condition using bright-field imaging. Their diameters were measured and they were counted in the selected region.

**Figure 3:**
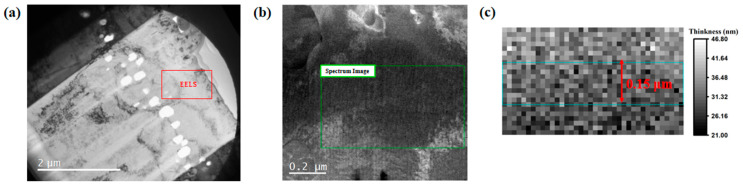
TEM images and EELS measurement of 15-15Ti after irradiation to a peak dose of 120 dpa. (**a**) The overview image under bright field. The red rectangle is the EELS measurement zone. (**b**) Dark-field image under the STEM mode in the helium-implanted zone. The dose is about 30 dpa in this zone. The green rectangle indicates the zone measured by the EELS method. (**c**) The actual thickness of the zone measured by EELS, obtained by multiplying the relative thickness from the EELS measurement with the mean free path of electrons. The green rectangle of width 150 nm indicates the selected region of cavity measurements.

In this paper, electron energy-loss spectroscopy (EELS) was employed to measure the local thickness of the TEM samples. EELS involves measurement of the energy distribution of electrons that have interacted with a specimen and lost energy due to inelastic scattering. It allows a quick and reliable measurement of local thickness in transmission electron microscopy. Local thickness can be calculated by $t/\lambda =\ln(I_{t}/I_{0})$, where *t* is the sample thickness, λ is the mean free path of electrons, and $I_{t}$ and $I_{0}$ are the integrated intensities of the total and zero energy loss peak, respectively. The λ values were measured to be 102, 104 and 98 nm for 200 keV electrons in Fe, Cr and Ni, respectively [42]. Then, the λ value for 15-15Ti could be obtained by weighting its main compositions, which gave 102 nm. A typical TEM image and the selected region where EELS was performed are shown in Figure 3a,b. The average thickness of the TEM lamella can be calculated at the same region as the cavity swelling measurement, as indicated in Figure 3c. 

**Figure 4:**
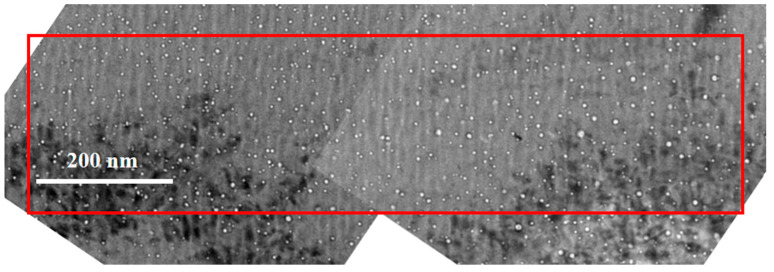
Combined TEM image for a specimen for which the dose of the helium implantation zone is 30 dpa. The red rectangle defines the zone where the cavities are measured. The TEM images were rotated so that the horizontal axis is parallel to the projected surface of the specimen.

The swelling caused by smaller cavities compared with the thickness of the TEM specimen can be calculated as follows:

(1) $$S=\frac{\Delta V}{V-\Delta V}$$

where ΔV is the region of cavities and V is the selected region of measurement. According to the procedure in Refs. [21,36], the cavity volume fraction is corrected due to the intersection of cavities with the surface, which is calculated by:

(2) $$\frac{\Delta V}{V}=\frac{1}{A}\sum_{i}\frac{\eta_{i}\Delta V\left(D_{i}\right)}{t-D_{i}}$$

where *A* and *t* are the area and the average thickness where the measurements are made and $D_{i}$ and $\eta_{i}$ are the diameter and the observed number of cavities in size class *i*. For multiple regions, the total cavity volume fraction can be written as follows:

(3) $$\frac{\sum_{k}\Delta V_{k}}{\sum_{k}V_{k}}=\frac{\sum_{k}\sum_{i}\eta_{k,i}\Delta V\left(D_{i}\right)t_{k}/\left(t_{k}-D_{i}\right)}{\sum_{k}A_{k}t_{k}}$$

where *k* indicates a different region. A typical image (the dose of the helium implantation zone is 30 dpa) with multiple TEM recordings is shown in Figure 4. The rectangle defines the region where the voids are evaluated. The width of that region is larger than 150 nm. The swelling of a specimen is given by the maximum value calculated with a band width of 150 nm. The swelling is readily calculated using Equation (1).

However, the swelling calculated by the above method will be seriously overestimated if the diameters of cavities are of a similar size to or have a greater thickness than the TEM sample. For cavities that cut completely through the TEM sample, the swelling can be calculated using $S=A_{V}/(A-A_{V})$, where $A_{V}$ is the region of cavities and *A* is the selected region of measurement.

### 2.3. The Correction of Damage Profile

The significant swelling induced by cavities will distort the distribution of displacement damage. The swelling is cumulated as cavities grow with time or dpa; therefore, it seems that the dpa profile should be corrected spatially and temporally. To account for the effect of cavity swelling on the damage profile, various correction methods were developed usually using reduced mass density in the SRIM calculations (see Refs. [16,43]). 

The fixed damage rate method and the fixed depth method were proposed in Ref. [43]. In the fixed damage rate method, the dpa profile is simply stretched according to the swelling profile. In the fixed depth method, the dpa profile is corrected by using the reduced density in SRIM calculations. The mass density of each bin is calculated from the swelling profile at the final state, i.e., the worst case, or the averaged swelling profile at the beginning and the end of the irradiation. In any case, the dpa peak would be the same in both methods. Authors have preferred the fixed depth method because the inaccurate overlapping of dpa, injected interstitial and pre-implanted helium could lead to unfair comparison between different doses. Kim also proposed a correction procedure by using a reduced mass density which evolves with swelling in the SRIM calculations [16]. However, the density correction formula used in Refs. [16,43] is an approximation to the accurate one. A small difference in dpa peaks before and after the correction is more likely due to this approximation error in the case of large swelling. It should be noted that not only the dpa profile but also the injected interstitial and helium profiles should be corrected, as they will be pushed to the deeper region in the case of swelling. 

**Figure 5:**
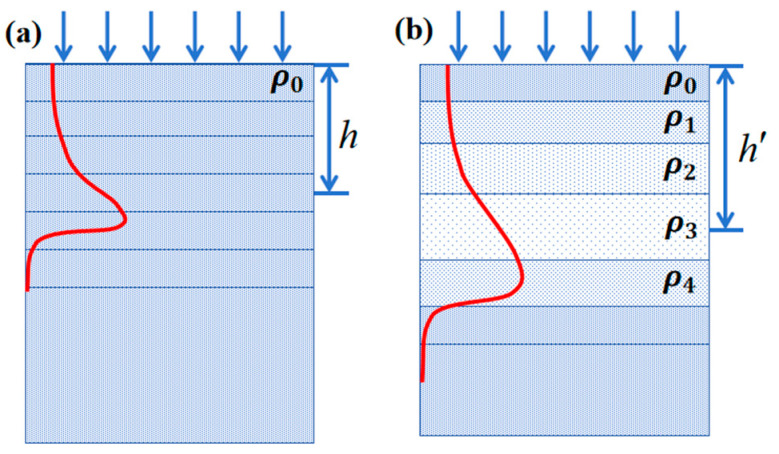
A schematic drawing of ion injection into a material with (**a**) normal and (**b**) reduced densities. Where $\rho_{0}$ is the original density and $\rho_{1}$, $\rho_{2}$, $\rho_{3}$ and $\rho_{4}$ are the reduced density. The bin with depth at *h* before irradiation is at *h*′ after irradiation.

In the following, we will re-derive the dpa correction formula using an accurate reduced density. The basic idea is that cavities do not cause any energy loss. The formalism is essentially the same as in the fixed damage rate method, as shown in Figure 5. 

Assume the unirradiated materials before the depth, *h*, are divided into *n* bins. Considering a bin at the depth of *h* before irradiation, because of the mass conservation, the mass density at the expanded depth, *h*′, reads:

(4) $$\rho^{\prime }(h^{\prime })=\frac{\rho V}{V+\Delta V}=\frac{\rho }{1+S(h^{\prime })}$$

where *S*(*h*′) is the local swelling at the expanded depth, *h*′. In the case of small swelling, the above equation can be approximated as $\rho^{\prime }(h^{\prime })=(1-S)\rho$, which is exactly the same as Equation (1) in Ref. [16]. However, it would be inaccurate for large swelling. Assuming that swelling during ion irradiation occurs along the irradiated direction, the mass conservation can also be written as follows:

(5) $$\rho dh=\rho^{\prime }(h^{\prime })dh^{\prime }=\frac{\rho }{1+S(h^{\prime })}dh^{\prime }.$$

By summing up all bins from the surface to *h*′, we obtain the following:

(6) $$h=\int_{0}^{h^{\prime }}\frac{1}{1+S(h^{\prime })}dh^{\prime }.$$

Assuming that the swelling is caused by cavities and that there is no energy loss in them, the number of displaced atoms per injected ion remains the same in each bin whether there is swelling or not; the same is true for the dpa rate or total dpa, i.e.:

(7) $$\xi^{\prime }(h^{\prime })=\xi (h)=\xi (\int_{0}^{h^{\prime }}\frac{1}{1+S(h^{\prime })}dh^{\prime })$$

where ξ(h) is the original dpa profile and $\xi^{\prime }(h^{\prime })$ is the corrected dpa profile. Now it is free to make a change of *h*′→*h*, so we obtain a correction formula for dpa. As for the concentration of injected interstitial and helium in a bin, because they are calculated as the ratio of deposited atoms to material atoms in this bin, similar to displaced atoms, Equation (7) can also be used to correct the concentration profile for injected interstitial and helium.

Equation (8) seems problematic because the swelling is a changing factor during the irradiation. In the following, we will prove that there is no need to divide time intervals in the correction. Assuming that the unirradiated materials before the depth, *h*, are divided into *n* bins and that the irradiation period is divided into *m* time intervals, *S_ij_* is the swelling for the *i*th bin during the *j*th time interval. The bin at depth *h* will be at *h*′ after the irradiation. It can be expressed by the sequential increment of depths in each time interval:

(8) $$h^{\prime }=h\prod_{j=1}^{m}\frac{h_{j}}{h_{j-1}}=h\prod_{j=1}^{m}\frac{\sum_{i=1}^{n}\frac{h}{n}\prod_{k=1}^{j}\left(1+S_{ik}\right)}{\sum_{i=1}^{n}\frac{h}{n}\prod_{k=1}^{j-1}\left(1+S_{ik}\right)}$$

where *h_j_* is the depth of the bin at the original *h* after the *j*th time interval and $\frac{h}{n}\prod_{k=1}^{j}(1+S_{ik})$ is the thickness of the ith bin after the *j*th time interval. It can be easily proved that Equation (8) can be rewritten as follows:

(9) $$h^{\prime }=h\frac{\sum_{i=1}^{n}\frac{h}{n}\prod_{k=1}^{m}\left(1+S_{ik}\right)}{\sum_{i=1}^{n}\frac{h}{n}}=\frac{h}{n}\sum_{i=1}^{n}\left[\prod_{k=1}^{m}\left(1+S_{ik}\right)\right]$$

Alternatively, Equation (9) can be written in a differential form, i.e.:

(10) $$dh^{\prime }=[1+S(h)]dh$$

where *S*(*h*) is the swelling of the bin at the original *h*. In practice, the swelling is measured at the final state of an irradiated sample. We should treat a change of swelling as a function of depth; then, we obtain the following:

(11) $$\frac{1}{\left[1+S(h^{\prime })\right]}dh^{\prime }=dh$$

Equation (11) is actually the same as Equation (6). In other words, the dpa profile can be corrected by stretching the dpa profile using the depth-dependent swelling data. This can be understood by a schematic drawing in Figure 5. Based on the fact that cavities do not cause any energy loss, the damage rate is actually the same at *h* and *h*′ for the unirradiated and irradiated samples. It is also valid if reduced density for each layer is used in SRIM calculations.

## 3. Results and Discussion

**Figure 6:**
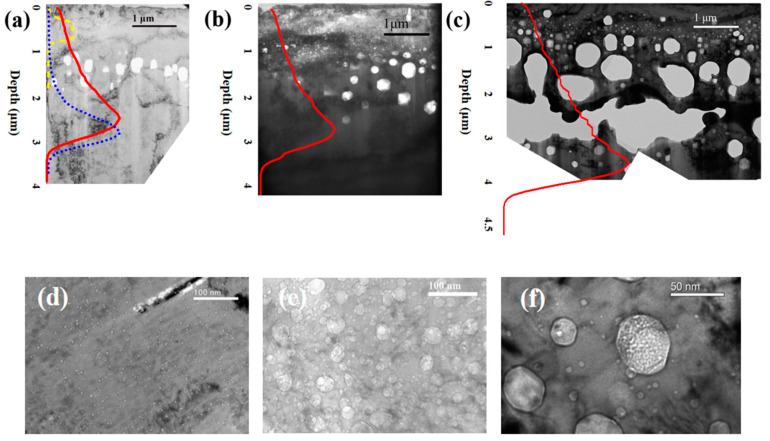
Microstructures of 15-15Ti after irradiation at peak doses of (**a**) 120 dpa, (**b**) 240 dpa and (**c**) 400 dpa. The images are overlapped with profiles of displacement damage (red solid lines) and implanted Ni (blue dotted line) and He (yellow dashed line). The damage curves as a function of depth in (**b**,**c**) were corrected due to the existence of extraordinarily large cavities. The microstructures in the helium-implanted zone are shown in (**d**–**f**) with doses of 30 dpa, 60 dpa and 122 dpa, respectively.

TEM images of irradiated samples with different dpa values are shown in Figure 6, overlapped with the damage curves. Small cavities can be seen in the helium-implanted zone around a depth of 1 μm (Figure 6d–f), while large cavities can be observed in the deeper regions, which are beyond the helium implantation ranges (see Figure 6a–c). Particularly for the sample with the peak dose of 400 dpa, the cavities coalesced to a very large cavity near the damage peak [44]. No cavity was observed at the damage peak. It is to be noted that the size of cavities generally increases with increasing dose in both helium-implanted and helium-free regions. Swelling did not occur in the peak damage region, indicating that the injected Ni ions suppressed swelling at the damage peak during irradiation [45,46]; in other words, the local vacancy supersaturation was lower than the threshold for cavity swelling.

Swelling was not observed in 15-15Ti by single-beam irradiation with nickel even if the irradiation dose was over 100 dpa [47]. However, large swelling was observed in the region that was presumably helium-free. In Ref. [48], it was shown that the helium could diffuse further into a deeper region due to the vacancies created by ion bombardment. Those helium atoms could enhance the cavity nucleation, which can lead to significant swelling when combined with large doses.

Based on the fact that cavities do not cause any energy loss of injected ions, a dpa correction formula was re-derived in Section 2.3. The dpa profile is like stretching the original curve for an irradiated sample. In order to perform the dpa profile correction, the TEM images were divided into five bins of equal width and the swelling of each bin was measured. The depth-dependent swelling was then interpolated from the data of the sectioned bins. Using Equation (7) in Section 2.3, the damage curve was corrected and is shown in Figure 6b,c overlapped with the TEM image.

Compared with the helium-implanted zone and the region with large cavities, the size of the cavities is smaller and the number density is larger in the helium-implanted zone than in the displacement-damage-only region under the same dose. Note that the helium implantation zone in Figure 6b and the region just beyond the helium implantation range in Figure 6a receive a similar dose of 70 dpa. However, the cavities in the latter region are significantly larger than those in the helium zone, indicating that the implanted helium may play a role in the growth of cavities. The abnormality can be found by comparing two regions in Figure 6b,c, where the irradiation dose is around 122 dpa.

**Figure 7:**
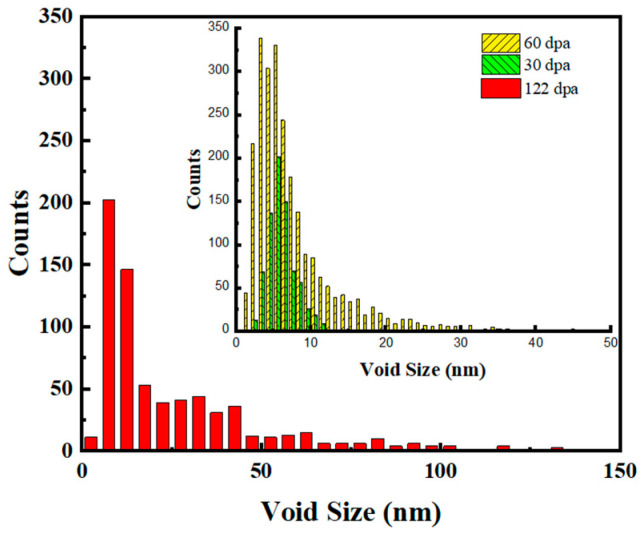
Cavity size distribution in the helium-implanted zone.

The cavities in the helium-implanted zones were measured and counted at different doses, with their size distribution shown in Figure 7. It can be seen that the tail of the size distribution increases markedly with increasing dpa, although the peak shifts only moderately to the higher size, from ~5.5 nm to 7.5 nm, as the damage increases from ~30 dpa to ~122 dpa. Much larger cavities over a hundred nanometers were seen in the helium implantation zone as the damage reached ~122 dpa.

**Figure 8:**
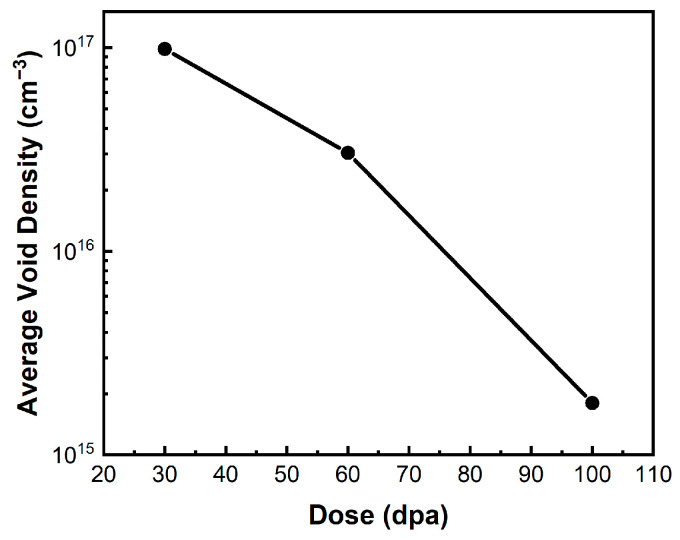
Cavity densities in the helium-implanted zone.

The cavity density in the helium-implanted zones is compared in Figure 8. It can be seen that the cavity density decreased significantly as the dose increased. A previous study has shown that voids rather than bubbles predominate in specimens after helium implantation followed by high-dose heavy ion irradiation [21]. The increase in cavity size and the decrease in density are mainly due to the growth and coalescence of cavities with increasing dose.

**Figure 9:**
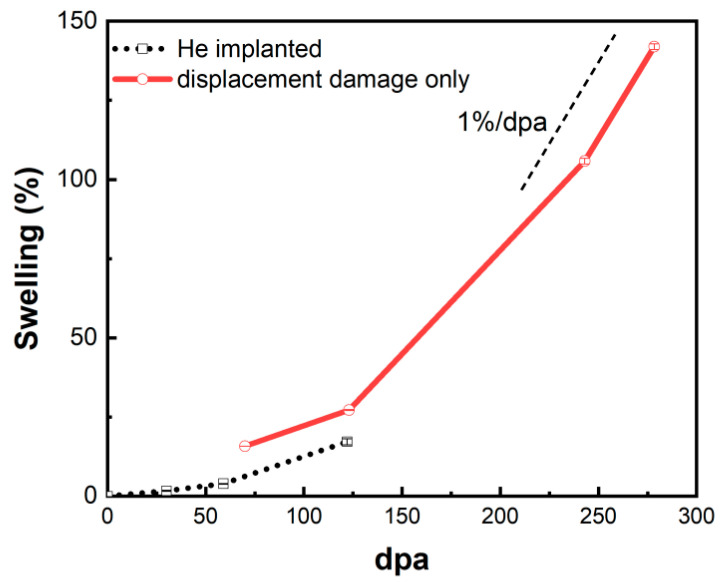
Cavity swelling of He-implanted zone and near the damage peak region.

The cavity swelling in the helium-implanted zone and the helium-free region near the damage peak as a function of dpa were drawn in Figure 9. It can be seen that the swelling is more than 100% at 243 dpa and increases up to 142% at 278 dpa. As discussed in the previous paragraphs, the swelling increases with the increasing dose in these two regions. In the helium-free region, it can be found that a steady-state swelling rate reaches ~1%/dpa at high doses, consistent with the data of austenitic stainless steels irradiated by neutron irradiation. However, it can be seen more clearly that the swelling in the helium-implanted zone is lower than that near the damage peak region in the range of 70–122 dpa. In the dislocation bias model, swelling is caused by bias-driven vacancy growth, which requires excess vacancy flux [49]. Cavities such as helium bubbles become the main point defect sinks due to the increase in density in the presence of excessive helium. Plentiful cavities as neutral sinks result in nearly equal absorption of interstitials and vacancies, thereby inhibiting cavity growth and suppressing swelling [25].

From the above discussion, it can be seen that the ion irradiation combined with helium pre-implantation reproduced an incubation period, a transient regime and, finally, the steady state of swelling. The swelling evolution and the steady-state swelling rate of 1%/dpa are similar to the swelling behavior of austenitic steels irradiated in reactors. This study provides a successful example of using ion irradiation to simulate neutron irradiation. With careful design of experiments, the ion irradiation technique can be very useful not only in the study of neutron irradiation effects but also in the quick screening of irradiation-resistant materials.

## 4. Conclusions

In summary, the swelling behavior of 15-15Ti stainless steel was investigated by pre-implantation of helium at room temperature followed by Ni ion irradiation at 580 °C to peak doses of 120, 240 and 400 dpa. TEM lamellas were prepared and observed for the irradiated samples. Electron energy loss spectroscopy was used to measure the thickness of the regions with cavities in the TEM lamellas. The swelling induced by smaller-sized cavities was calculated based on the cavity and thickness measurements in the same region. Large cavities were observed in the region with displacement damage only and coexisted with smaller cavities in the helium-implanted zone. The swelling in the region of large cavities was determined by measuring the cavity area ratio of the porous region. A correction formula of the dpa curve was proposed using more accurate reduced densities and based on the fact that the cavities do not cause any energy loss.

The size of cavities increases but the density decreases with increasing dose in both helium-implanted and helium-free regions, which is mainly attributed to the growth and coalescence of cavities under irradiation. 15-15Ti shows severe swelling at high doses, with swelling exceeding 100% at 243 dpa. The steady-state swelling rate of ~1% /dpa was observed in 15-15Ti at high doses, consistent with that in austenitic stainless steels by neutron irradiation.

Comparing the swelling in the helium-implanted and helium-free regions, the size of cavities was much smaller in the helium-implanted zone than in the damage region at the same doses, 70 and 122 dpa, although the number density was larger. A lower swelling was observed in the helium-implanted zone. It seems that excessive helium will suppress swelling at such doses. The reason behind this may be that the higher cavity density in the helium-implanted zone promotes the recombination of interstitials and vacancies, thus inhibiting the growth of cavities.

This study provides a successful example of using ion irradiation to simulate the neutron irradiation effect. And it is hoped that the proposed formula will resolve the controversy over the correction of the dpa curve due to swelling for an ion-irradiated sample. However, there are still some ambiguities regarding the role of helium in irradiation swelling, especially its dynamic behavior in response to ion bombardment. The dynamic behavior and the effect of helium concentration on ion irradiation swelling should be investigated in the future.

## Data Availability

Data are contained within the article.

## Abbreviations

CIAE	China Institute of Atomic Energy
dpa	displacements per atom
EELS	Electron energy-loss spectroscopy
FIB	Focused ion beam
PBN/PG	Pyrolytic boron nitride/pyrolytic graphite
SFR	Sodium-Cooled Fast Reactor
SS	Stainless steel
TEM	Transmission electron microscopy
